# Changes in glucose metabolism, C-reactive protein, and liver enzymes following intake of NAD + precursor supplementation: a systematic review and meta‐regression analysis

**DOI:** 10.1186/s12986-024-00812-0

**Published:** 2024-06-24

**Authors:** Mohammad Hassan Sohouli, Sogand Tavakoli, Marcela Gomes Reis, Azita Hekmatdoost, Nathalia Sernizon Guimarães

**Affiliations:** 1 Student Research Committee, Department of Clinical Nutrition and Dietetics, Faculty of Nutrition and Food Technology, Shahid Beheshti University of Medical Sciences, Tehran, Iran; 2 Health Science at Faculdade, Ciências Médicas de Minas Gerais, Belo Horizonte, Minas Gerais Brazil; 3 Department of Clinical Nutrition and Dietetics, Faculty of Nutrition and Food Technology, Shahid Beheshti University of Medical Sciences, Tehran, Iran; 4 Department of Nutrition, Universidade Federal de Minas Gerais, Belo Horizonte, Minas Gerais 30130-100 Brazil

**Keywords:** NAD + precursor, Niacin, Glucose metabolism, CRP, Liver enzyme, Insulin resistance, Meta analysis

## Abstract

**Background:**

There are contradictory effects regarding the effect of NAD + precursor on glucose metabolism and liver enzymes. In order to obtain a better viewpoint from them, this study aimed to comprehensively investigate the effects of NAD + precursor supplementation on glucose metabolism, C-reactive protein (CRP), and liver enzymes.

**Methods:**

PubMed/MEDLINE, Web of Science, SCOPUS, and Embase databases were searched using standard keywords to identify all controlled trials investigating the glucose metabolism, CRP, and liver enzymes effects of NAD + precursor. Pooled weighted mean difference (WMD) and 95% confidence intervals (95% CI) were achieved by random-effects model analysis for the best estimation of outcomes.

**Results:**

Forty-five articles with 9256 participants’ were included in this article. The pooled findings showed that NAD + precursor supplementation had a significant increase in glucose (WMD: 2.17 mg/dL, 95% CI: 0.68, 3.66, *P* = 0.004) and HbA1c (WMD: 0.11, 95% CI: 0.06, 0.16, *P* < 0.001) as well as a significant decrease in CRP (WMD: -0.93 mg/l, 95% CI -1.47 to -0.40, *P* < 0.001) compared with control group, and was not statistically significant with respect to insulin and homeostasis model assessment of insulin resistance (HOMA-IR). However, we found no systemic changes in aspartate transaminase (AST), alanine transaminase (ALT), or alkaline phosphatase (ALP) levels after NAD + precursor supplementation. The results of the subgroup analysis showed that the intake of NAD + precursor during the intervention of more than 12 weeks caused a greater increase in the glucose level. Furthermore, Nicotinic acid supplementation (NA) causes a greater increase in glucose and HbA1c levels than nicotinamide (NE) supplementation.

**Conclusions:**

Overall, these findings suggest that NAD + precursor supplementation might have an increase effect on glucose metabolism as well as a decrease in CRP.

**Supplementary Information:**

The online version contains supplementary material available at 10.1186/s12986-024-00812-0.

## Introduction

Diabetes is a chronic and progressive disease generally characterised by high fasting blood glucose concentrations [1, 2] or changes in levels of other factors such as glycated haemoglobin (HbA1c) [3, 4], and HOMA-IR [5]. Evidence suggests that diabetes impairs the function of different organs like the heart, kidneys, eyes, and especially the liver [6–10]. In actuality, diabetes causes liver function to be disrupted due to the increased lipid influx into the liver and de novo lipid syntheses [11, 12], which are shown by elevated liver enzymes (AST and ALT) [13]. In addition, diabetes causes disruption in mitochondrial function [14], metabolic dysregulation [15], oxidative damage [16], and NAD + redux abnormalities [17–19].

According to estimates, the number of individuals over 20 years old with diabetes will rise to more than 700 million by 2045 [20]. Therefore, conducting interventional studies in order to stop the complications of diabetes seems necessary.

The NAD + precursor and related compounds are of great interest due to their therapeutic effects, especially in the treatment of hyperlipidemia [21]; the findings indicate that the NAD + precursor, which is predominantly synthesised by the salvage pathways from the recovery of nicotinamide (NE) and nicotinic acid (NA) biogenesis, is an essential metabolic cofactor in cellular metabolism [22]. Thus, maintaining the cytosolic NAD^+^/NADH ratio within the normal range is critical. While this ratio decreases in diabetes and is referred to as pseudohypoxia, which leads to oxidative stress [19, 23]. Yoshinno et al. also reported the depletion of NAD + in mice liver due to the accumulation of fat caused by insulin resistance and impaired glucose metabolism [24]. In recent years, studies have shown that NAD + precursors can significantly cause hyperglycemia and reduce inflammation [18, 21] and have a moderate effect on liver enzymes [25, 26]. However, the NAD + supplementation effect on glucose metabolism measurement criteria and liver enzymes is still obscure. In order to assess the effects of different NAD + precursor supplements on fasting blood glucose, HbA1c, insulin, and HOMA-IR as well as CRP, ALT, AST, and ALP as liver enzymes, this systematic review and meta-regression analysis based on known clinical studies was conducted.

## Methods

### Search strategy

The Preferred Reporting Items for Systematic Review and Meta-analysis (PRISMA) criteria were followed for conducting this study [27]. Without regard to language or time restrictions, a thorough search was carried out in the PubMed/MEDLINE, Web of Science, SCOPUS, and Embase databases from the beginning to April 2024. Additionally, similar papers and gray literature were considered in the search. Medical subject headings (MeSH) and Emtree (Embase subject headings) were selected to search the online databases, as follow: (“NAD” OR "NAD precursor" OR "Nicotinic Acids" OR "Niacin" OR "Niacinamide" OR "Nicotinamide Mononucleotide" OR Niaspan OR acipomax OR Niagen) AND (“Insulin Resistance” OR Insulin OR HOMA-IR OR Glucose OR “Glucose Intolerance” OR “Glycated Hemoglobin” OR “HbA1c” OR "C-Reactive Protein" OR "Inflammation" OR “Aspartate Transaminase” OR AST OR “Alanine Transaminase” OR ALT OR SGOT OR SGPT OR “Alkaline Phosphatase” OR ALP) AND ("Clinical Trials as Topic" OR "Cross-Over Studies" OR "Double-Blind Method" OR "Single-Blind Method" OR "Random Allocation" OR "Clinical Trial") (The specific search strategy is described in the Supplementary Appendix S1). The reference lists of the publications retrieved and linked review studies were manually searched to identify potentially overlooked qualifying trials. We also performed a “snowball search” to add other RCTs (not included in this study).

### Eligibility criteria

Using titles, abstracts, or the complete texts of the research, two writers separately removed duplicate articles before finding and reviewing relevant publications. In the end, the papers were separated based on the following standards: 1) Randomized clinical trials studies; 2) NAD + precursor supplementation (nicotinic acid (NA) or nicotinamide (NE) supplementation) was given as an intervention in individual’s aged 18 and over; and 3) baseline and post in both group (intervention and control) glucose, insulin, HOMA-IR, HbA1c, CRP, ALP, AST, and ALT were recorded. The most recent or longest follow-up period was used when a research revealed results at more than one follow-up time. Studies with duplicated data, studies with ambiguous information, studies in which NAD + precursor was used as an intervention alongside other commonly prescribed medications, non-randomized trial designs, animal studies, studies without a control group, reviews, and meta-analysis studies were also excluded. The PICOS criteria for inclusion and exclusion of studies were as follows. Population: individual’s aged 18 and over; Intervention: NAD + precursor supplementation (nicotinic acid (NA) or nicotinamide (NE) supplementation); Comparator: other intervention or placebo; Outcomes: glucose, insulin, HOMA-IR, HbA1c, CRP, ALP, AST, and ALT; Study design: randomized clinical trials studies.

### Data extraction

The qualifying studies were examined by two authors independently. The first author's name, the study's location, the year it was published, the sample size (for the intervention and control groups), the participant characteristics (such as the percentage of men, age, and health status), the type of outcomes, duration of the intervention, the dosage and type of the intervention, and the means and standard deviations (S.D.s) of the intended outcomes at baseline, post-intervention, and/or changes between baseline and post-intervention, were all extracted.

### Quality assessment

Using the Cochrane risk-of-bias test for randomized trials (RoB 2), version 2, the quality of the included RCTs was methodologically evaluated [28]. Based on the following potential sources of bias: blinding of outcome assessment, allocation concealment, participant and staff blinding, random sequence generation, incomplete outcome data, selective reporting, and other bias, two authors independently rated each study as having a low, high, or unclear risk of bias. Any discrepancies were discussed with a third author in order to come to a consensus. The GRADE (Grading of Recommendations Assessment, Development, and Evaluation) grading method was also used to evaluate the quality of the current analytic research [29]. A reliable 10-point assessment system that assesses elements affecting study quality is the GRADE checklist. This scale has seven components: (1) risk of bias, (2) precision, (3) heterogeneity, (4) directness, (5) publishing bias, (6) funding bias, and (7) study design.

### Data synthesis and statistical analysis

The data were examined using STATA version 12.0 software. Different data types were converted using a predetermined procedure to the mean and standard deviations (S.D.s) [30, 31]. For instance, in the absence of standard deviations, we calculated the change using the method below: The definition of standard deviation changes is square root [(S.D. baseline ^2^ + SD final ^2^)—(2R S.D. baseline 2 S.D. final)]. The following formula is used to convert the standard error of the mean (SEM) to standard deviation: S.D. is equal to SEM × √n, where n is the total number of participants in each group. The random-effects model was employed in the meta-analysis of research results. R codes used for analysis is described in the Supplementary Appendix S2. The weighting of the research followed the typical inverse variance technique. The data from the longest time point were used for the analysis, which allowed for the handling of many assessments within a single study group. Using Q Statistics and I-squared (I^2^), the degree of study heterogeneity was evaluated. Insignificant, low, moderate, and high heterogeneity were found with I^2^ values ranging from 0% to 25, 26% to 50%, 5% to 75%, and 76% to 100%, respectively [32]. To identify possible causes of heterogeneity, a pre-defined subgroup analysis based on the dosage, duration, and type of the intervention was conducted. A sensitivity analysis was done to determine the contribution of each research to the overall mean difference. In order to establish if there was publication bias, we utilized the official Egger's test [33].

## Results

**Fig. 1:**
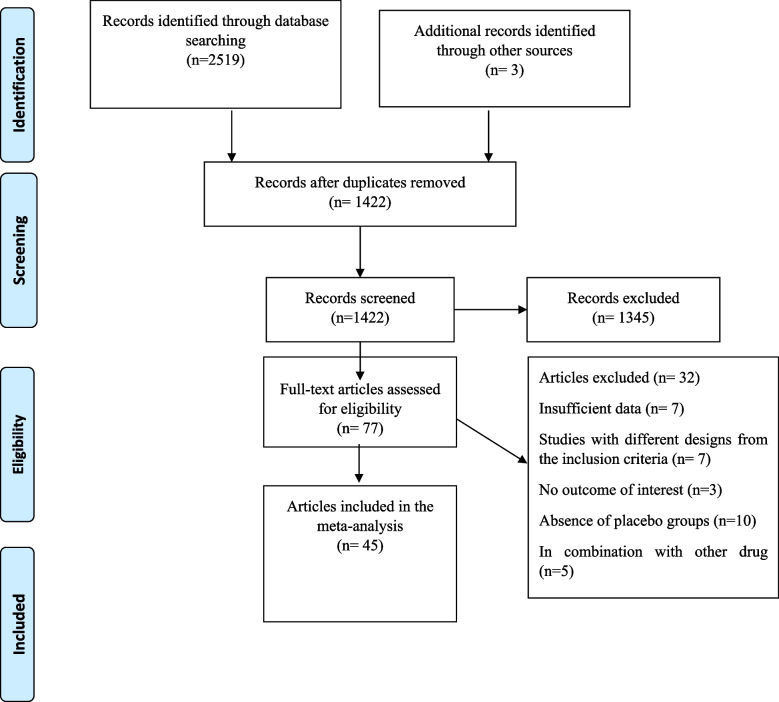
Flow chart of the study, including identification, screening, eligibility, and the final sample included

Figure 1 depicts a flowchart of the research selection process with exclusion criteria. This value indicates that the aforementioned electronic databases generated 2519 articles. After removing publications with duplicate research, there were 1422 total. Following an assessment of the research's titles and abstracts, 1345 papers were dropped since they didn't meet the inclusion requirements. 77 articles were found utilizing the full-text search during the secondary screening. For the reasons listed above, 32 of the investigations were dropped. Finally, 45 papers were included in the quantitative meta-analysis since they matched the qualifying requirements.

### Study characteristics

The features of the pooled articles are shown in Table 1. Our surveys reveal that 19 studies have been carried out in USA, 14 articles in the European continent, 11 studies in the Asia and one article in the Egypt. Also, a multicenter study was conducted. All articles were published between 1998 and 2022 and follow up intervention ranged from 4 to 144 weeks. The mean age and percentage of male participants ranged from 26.3 to 71.1 years and 0–100%, respectively, at the baseline. Six studies were conducted with crossover design and the rest of the study was conducted in parallel. The doses prescribed in the studies were between 100 and 3000 mg per day, and in nine studies the supplement type was in the form of NE and the rest were in the form of NA. In addition, the population investigated in the studies included people with diabetes or glucose tolerance disorder, non-alcoholic fatty liver disease, metabolic syndrome, obese people, polycystic ovary syndrome, people with dyslipidemia or cardiovascular problems, as well as healthy people.

**Table 1:** Characteristics of eligible studies

Author (year)	Country	Population	RCT design	Mean Age (year)	Sex (Male %)	Sample Size Study (intervention/control)	Follow up of intervention (Weeks)	Type of intervention	Dose (mg/day) of intervention	Type of Control Group	Outcomes
*Dollerup *et al. *2019 *[34]	*Denmark*	*Men With Obesity*	*Parallel*	*59*	*100*	*20/20*	*12*	*NE*	*2000*	*Placebo*	*Glucose, Insulin, HOMA-IR*
*Canner *et al. *2006 *[35]	*USA*	*Metabolic Syndrome and Healed Myocardial Infarction*	*Parallel*	*NR*	*NR*	*964/2468*	*48*	*NA*	*2000*	*Placebo*	*Glucose*
*Linke *et al. *2009 *[36]	*Germany*	*Patients with impaired glucose tolerance*	*Parallel*	*45.5*	*70*	*30/30*	*24*	*NA ( Extended-release)*	*1000*	*Individuals in the control group did receive any medication or lifestyle intervention*	*Glucose, HbA1c, Insulin, HOMA-IR, CRP*
*Villines *et al. *2010 *[37]	*USA*	*Patients with coronary heart disease*	*Parallel*	*65*	*NR*	*154/161*	*56*	*NA( Extended-release)* + *Ezetimibe (10 mg/day)*	*2000*	*Ezetimibe (10 mg/day)*	*Glucose, CRP*
*Guyton *et al. *2008 *[38]	*USA*	*Patients With Type IIa or Type IIb Hyperlipidemia*	*Parallel*	*56.9*	*41.9*	*381/207*	*24*	*NA( Extended-release)* + *Ezetimibe/Simvastatin (10/20 mg/day)*	*2000*	*Ezetimibe/Simvastatin (10/20 mg/day)*	*Glucose, CRP*
*Philpott *et al. *2013 *[39]	*Australia*	*Patients with established coronary artery disease*	*Cross over*	*57.9*	*83*	*66/66*	*12*	*NA( Extended-release)*	*1500*	*Placebo*	*Glucose, CRP*
*Fabbrini *et al. *2010 *[40]	*USA*	*NAFLD*	*Parallel*	*43*	*30*	*9/9*	*16*	*NA( Extended-release)*	*2000*	*Placebo*	*Glucose, Insulin, HOMA-IR*
*Abdi *et al. *2007 *[41]	*Iran*	*Dyslipidemic patients*	*Cross over*	*56.45*	*64.1*	*50/50*	*6*	*NA*	*200*	*Placebo*	*Glucose, ALP, AST, ALT*
*Aye *et al. *2014 *[42]	*UK*	*Polycystic ovary syndrome*	*Parallel*	*31*	*0*	*13/12*	*12*	*NA*	*1000*	*Placebo*	*Glucose, HbA1c, Insulin, HOMA-IR, CRP*
*Huang *et al. *2022 *[43]	*India*	*Healthy subjects*	*Parallel*	*47.76*	*41.9*	*31/31*	*8*	*NE*	*300*	*Placebo*	*Glucose, Insulin, AST, HOMA-IR, ALP, ALT*
*Bregar *et al. *2014 *[44]	*Slovenia*	*Patients after myocardial infarction*	*Parallel*	*52.5*	*100*	*33/30*	*12*	*NA( Extended-release)*	*2000*	*Placebo*	*Glucose*
*Hamilton *et al. *2010 *[45]	*Australia*	*T2DM*	*Parallel*	*65*	*NR*	*7/8*	*12*	*NA( Extended-release)*	*1500*	*Individuals in the control group did receive any medication or lifestyle intervention*	*Glucose, HbA1c*
*Dollerup *et al. *2018 *[46]	*Denmark*	*Obese men*	*Parallel*	*58*	*100*	*20/20*	*12*	*NE*	*2000*	*Placebo*	*Glucose, HbA1c, ALT*
*Westphal *et al. *2007 *[47]	*Germany*	*MetS*	*Parallel*	*55*	*100*	*20/10*	*6*	*NA( Extended-release)*	*1500*	*Placebo*	*Glucose, HbA1c, Insulin, HOMA-IR, CRP*
*Vittone *et al. *2007 *[48]	*USA*	*MetS*	*Parallel*	*54*	*86.2*	*80/80*	*144*	*NA*	*2000*	*Placebo*	*Glucose, Insulin*
*Thoenes *et al. *2007 *[49]	*USA*	*MetS*	*Parallel*	*34.6*	*56*	*30/15*	*52*	*NA( Extended-release)*	*1000*	*Placebo*	*Glucose, CRP*
*Taylor *et al. *2006 *[50]	*USA*	*Patients with coronary heart disease*	*Cross over*	*67*	*92.3*	*57/47*	*96*	*NA( Extended-release)*	*1000*	*Placebo*	*Glucose, CRP*
*Sorrentino *et al. *2010 *[51]	*Switzerland*	*T2DM*	*Parallel*	*60*	*84.8*	*15/15*	*12*	*NA( Extended-release)*	*1500*	*Placebo*	*Glucose, HbA1c*
*Savinova *et al. *2015 *[52]	*USA*	*MetS*	*Parallel*	*47*	*57*	*14/14*	*16*	*NA( Extended-release)*	*2000*	*Placebo*	*Glucose, HbA1c*
*Chow *et al. *2010 *[53]	*USA*	*HIV-infected patients*	*Parallel*	*50*	*89*	*10/9*	*12*	*NA( Extended-release)*	*1500*	*Individuals in the control group did receive any medication or lifestyle intervention*	*Glucose, Insulin, HOMA-IR, CRP*
*Goldberg *et al. *2016 *[54]	*USA*	*Nondiabetic statin-treated subjects*	*Parallel*	*62.9*	*85*	*274/264*	*144*	*NA( Extended-release)*	*1500*	*Placebo*	*Glucose, Insulin, HOMA-IR*
*Nash *et al. *2011 *[55]	*USA*	*Chronic tetraplegia*	*Parallel*	*32.2*	*NR*	*31/23*	*48*	*NA( Extended-release)*	*2000*	*Placebo*	*Glucose, HbA1c*
*Warnholtz *et al. *2009 *[56]	*Germany*	*Patients with coronary heart disease*	*Parallel*	*65*	*94*	*53/53*	*12*	*NA( Extended-release)*	*1000*	*Placebo*	*Glucose, Insulin*
*Osar *et al. *2004 *[57]	*Turkey*	*Patients with Poorly Controlled T2DM*	*Parallel*	*58*	*46.6*	*15/15*	*4*	*NE*	*3000*	*Placebo*	*Glucose, HbA1c, CRP*
*Kei *et al. *2013 *[58]	*Greece*	*Mixed dyslipidaemia*	*Parallel*	*58*	*53.8*	*26/32*	*12*	*NA* + *Rosuvastatin (40 mg)*	*2000*	*Rosuvastatin (40 mg)*	*Glucose, CRP, AST, ALT*
*Elam *et al. *2000 *[59]	*Multi center*	*Diabetes and peripheral arterial diseases*	*Parallel*	*67*	*87.2*	*61/59;169/161*	*60*	*NA( Extended-release)*	*3000*	*Placebo*	*Glucose, HbA1c*
*Igarashi *et al. *2022 *[60]	*Japan*	*Healthy older men*	*Parallel*	*71.1*	*100*	*21/21*	*12*	*NE*	*250*	*Placebo*	*Glucose, HbA1c, HOMA-IR*
*Otto *et al. *1998 *[61]	*Germany*	*Mixed dyslipidaemia*	*Cross over*	*49.3*	*61.1*	*18/18*	*24*	*NA*	*600*	*Placebo*	*Glucose, ALP, AST, ALT*
*Ko *et al. *1998 *[62]	*Hong Kong*	*Diabetes*	*Cross over*	*59.2*	*36.3*	*32/30*	*12*	*NA* + *Lovastatin (40 mg)*	*750*	*Lovastatin (40 mg)*	*Glucose, HbA1c, ALT*
*Song *et al. *2019 *[63]	*Korea*	*Patients with high level of lipoprotein*	*Parallel*	*65*	*44*	*13/18*	*96*	*NE*	*1000*	*Individuals in the control group did receive any medication or lifestyle intervention*	*HbA1c*
*Kang *et al. *2013 *[64]	*Korea*	*Patients with chronic kidney disease*	*Parallel*	*55.8*	*38.7*	*31/30*	*24*	*NA* + *Statin*	*500*	*Statin*	*HbA1c, CRP, ALP, AST, ALT*
*Lee *et al. *2009 *[65]	*UK*	*Patients with coronary artery disease*	*Parallel*	*65*	*94*	*22/29*	*48*	*NA(modified-release nicotinic acid)*	*2000*	*Placebo*	*CRP, HbA1c*
*Bays *et al. *2010 *[66]	*USA*	*Dyslipidemic patients with Mets*	*Parallel*	*57.7*	*62.4*	*221/110*	*24*	*NA( Extended-release)*	*2000*	*Placebo*	*HbA1c*
*Owada *et al. *2003 *[67]	*Japan*	*Chronic Renal Disease*	*Parallel*	*57*	*50*	*16/17*	*60*	*NA*	*1500*	*Individuals in the control group did receive any medication or lifestyle intervention*	*HbA1c*
*Okabe *et al. *2022 *[68]	*Japan*	*Healthy Subjects*	*Parallel*	*42.9*	*26.6*	*15/15*	*16*	*NE*	*250*	*Placebo*	*ALP, AST, ALT*
*El-Kady *et al. *2022 *[69]	*Egypt*	*NAFLD*	*Parallel*	*45.6*	*41.9*	*31/30*	*12*	*NA* + *Antidiabetic therapy*	*1000*	*Antidiabetic therapy*	*HOMA-IR, AST, ALT*
*Goldberg *et al. *2000 *[70]	*USA*	*Patients with primary hyperlipidemia*	*Cross over*	*54*	*57*	*87/44*	*24*	*NA*	*3000*	*Placebo*	*AST, ALT*
*Moore *et al. *2007 *[71]	*USA*	*Atherosclerotic disease*	*Parallel*	*54.7*	*71*	*42/41*	*60*	*NA( Extended-release)* + *Atorvastatin*	*100*	*Atorvastatin*	*AST, ALT*
*Conze *et al. *2019 *[72]	*USA*	*Healthy overweight Adults*	*Parallel*	*52.3*	*34*	*33/34*	*8*	*NE*	*2000*	*Placebo*	*AST, ALT*
*Vidal *et al. *2000 *[73]	*Spain*	*Type 1 Diabetes*	*Parallel*	*26.3*	*54.5*	*11/12*	*60*	*NE* + *Atorvastatin*	*2100*	*Atorvastatin*	*HbA1c*
*Airan-Javia *et al. *2009 *[74]	*USA*	*Patients with carotid atherosclerosis*	*Parallel*	*71*	*72*	*26/25*	*48*	*NA( Extended-release)* + *Simvastatin (20 mg)*	*2000*	*Simvastatin (20 mg)*	*CRP*
*Shah *et al. *2010 *[75]	*USA*	*Patients with primary hypercholesterolaemia*	*Parallel*	*60.4*	*50.2*	*572/595*	*12*	*NA( Extended-release)* + *Statin (simvastatin 10 or 20 mg or atorvastatin 10 mg)*	*1000*	*Statin (simvastatin 10 or 20 mg or atorvastatin 10 mg)*	*CRP*
*Karacaglar *et al. *2015 *[76]	*Turkey*	*Acute coronary syndrome*	*Parallel*	*63*	*64*	*25/23*	*4*	*NA( Extended-release)* + *Statin*	*500*	*Statin*	*CRP*
*Fazio *et al. *2010 *[77]	*USA*	*Hyperlipidaemic patients*	*Parallel*	*57.4*	*59.2*	*391/212*	*64*	*NA( Extended-release)* + *Ezetimibe/Simvastatin (10/20 mg/day)*	*2000*	*Ezetimibe/Simvastatin (10/20 mg/day)*	*CRP*
*Lee *et al. *2011 *[78]	*Korea*	*Patients With Mild to Moderate Coronary Artery Stenosis*	*Parallel*	*58.1*	*50*	*14/14*	*36*	*NA* + *Smvastatin 40 mg*	*1000*	*Simvastatin 40 mg*	*CRP*

The findings of the evaluation of the eligible studies' quality are shown in Table 2. Additionally, a score of 7.6 (very good quality) was determined after the GRADE score system was used to assess the quality of the current meta-analysis. The Kappa result for the authors of our study for data screening and selection was about 0.92, which was interpreted as almost perfect agreement.

**Table 2:** Risk of bias assessment according to the Cochrane collaboration’s risk of bias assessment tool

Study, Year (reference)	Random sequence generation	Allocation concealment	Blinding of participants and personnel	Blinding of outcome assessment	Incomplete outcome data	Selective reporting	Overall assessment of risk of bias
Dollerup et al. 2019 [34]	Low	Low	Low	Low	Unclear	Low	Low
Canner et al. 2006 [35]	Low	Unclear	Low	Low	Unclear	Low	Unclear
Linke et al. 2009 [36]	Low	Low	Low	High	Unclear	Low	Unclear
Villines et al. 2010 [37]	Low	Low	Low	Low	Unclear	Low	Low
Guyton et al. 2008 [38]	Low	Unclear	Low	Low	Unclear	Low	Unclear
Philpott et al. 2013 [39]	Low	Low	Low	Low	Unclear	Low	Low
Fabbrini et al. 2010 [40]	Low	High	Low	Low	Unclear	Low	Low
Abdi et al. 2007 [41]	Low	Low	High	Low	Unclear	Low	Unclear
Aye et al. 2014 [42]	Low	Low	Unclear	Low	Unclear	Low	Low
Huang et al. 2022 [43]	Low	Unclear	Low	Low	Unclear	Low	Unclear
Bregar et al. 2014 [44]	Low	Low	Low	Low	Unclear	Low	Low
Hamilton et al. 2010 [45]	Low	Unclear	Unclear	Low	Unclear	Low	Low
Dollerup et al. 2018 [46]	Low	High	High	Low	High	Low	High
Westphal et al. 2007 [47]	Low	Low	High	Unclear	Unclear	Low	Unclear
Vittone et al. 2007 [48]	Low	Low	Unclear	Unclear	Unclear	Low	Unclear
Thoenes et al. 2007 [49]	Low	Low	High	Low	Unclear	Low	Unclear
Taylor et al. 2006 [50]	Low	Low	Unclear	Low	Unclear	Low	Low
Sorrentino et al. 2010 [51]	Low	Low	Low	Low	Unclear	Low	Low
Savinova et al. 2015 [52]	Low	High	Low	Low	Unclear	Low	Low
Chow et al. 2010 [53]	Low	Unclear	Low	High	Unclear	Unclear	Unclear
Goldberg et al. 2016 [54]	Low	Low	Low	Low	Unclear	Low	Low
Nash et al. 2011 [55]	Low	Low	Unclear	Low	Unclear	Low	Low
Warnholtz et al. 2009 [56]	Low	Unclear	Low	Low	Unclear	Low	Unclear
Osar et al. 2004 [57]	Low	Low	Low	Low	Unclear	Low	Low
Kei et al. 2013 [58]	Low	Unclear	Unclear	Low	Unclear	Low	Low
Elam et al. 2000 [59]	Low	Low	Low	High	Unclear	Low	Unclear
Igarashi et al. 2022 [60]	Low	Low	Low	Low	Unclear	Low	Low
Otto et al. 1998 [61]							
Ko et al. 1998 [62]	Low	Unclear	Low	Low	Unclear	Low	Unclear
Song et al. 2019 [63]	Low	Low	Low	Low	Unclear	Low	Low
Kang et al. 2013 [64]	Low	High	Low	Low	Unclear	Low	Low
Lee et al. 2009 [65]	Low	Unclear	Low	Low	Unclear	Low	Unclear
Bays et al. 2010 [66]	Low	Unclear	Low	Low	Unclear	Low	Unclear
Owada et al. 2003 [67]	Low	Low	Low	High	Unclear	Low	Unclear
Okabe et al. 2022 [68]	Low	Unclear	Low	Low	Unclear	Low	Unclear
El-Kady et al. 2022 [69]	Low	High	Low	Low	Unclear	Low	Low
Goldberg et al. 2000 [70]	Low	Low	Low	Low	Unclear	Low	Low
Moore et al. 2007 [71]	Low	Unclear	Low	Low	Unclear	Low	Unclear
Conze et al. 2019 [72]	Low	Low	Low	Low	Unclear	Low	Low
Vidal et al. 2000 [73]	Low	Low	Low	Low	Unclear	Low	Low
Airan-Javia et al. 2009 [74]	Low	Unclear	Low	Low	Unclear	Low	Unclear
Shah et al. 2010 [75]	Low	Low	Low	Low	Unclear	Low	Low
Karacaglar et al. 2015 [76]	Low	Unclear	Unclear	Low	Unclear	Low	Low
Fazio et al. 2010 [77]	Low	Low	Low	High	Unclear	Low	Unclear
Lee et al. 2011 [78]	Low	Low	Low	High	Unclear	Low	Unclear

#### *Meta*-analysis results

##### The effect of NAD + precursor supplementation on glucose metabolism

**Fig. 2:**
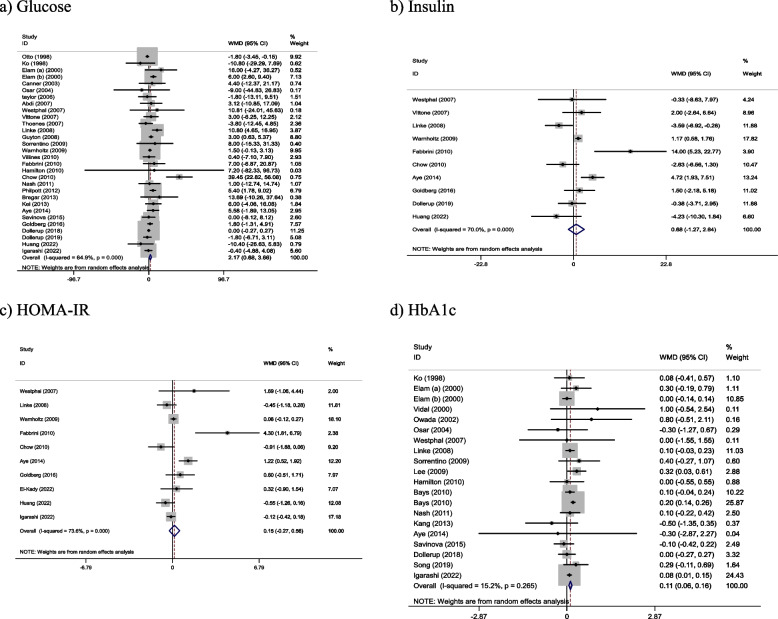
Forest plot of randomized controlled trials investigating the effects of NAD + precursor supplementation on (**a**) Glucose, (**b**) Insulin, (**c**) HOMA-IR, (**d**) HbA1c

With the use of random effects model, the pooled results indicated that NAD + precursor supplementation had a significant increased on glucose (WMD: 2.17 mg/dL, 95% CI: 0.68, 3.66, *P* = 0.004) and HbA1c (WMD: 0.11, 95% CI: 0.06, 0.16, *P* < 0.001) compared with control. However, compared to the control group, no significant effect on insulin (WMD: 0.68 μU/mL, 95% CI: -1.27, 2.64, *P* = 0.493) and HOMA-IR (WMD: 0.15, 95% CI: -0.27, 0.56, *P* = 0.488) was reported after receiving NAD + precursor. A high heterogeneity was shown in the trials for glucose (Cochran Q test, *P* < 0.001, I^2^ = 59.2–70.3%), insulin (Cochran Q test, *P* < 0.001, I^2^ = 67.1–73.9%) and HOMA-IR (Cochran Q test, *P* < 0.001, I^2^ = 69.6–77.8%). Although low heterogeneity was observed for HbA1c (Cochran Q test, *P* = 0.265, I^2^ = 11.4–17.1%) (Fig. 2).

#### Subgroups analysis

The results of the subgroup analysis showed that the intake of NAD + precursor during the intervention of more than 12 weeks caused a greater increase in the glucose level. Furthermore, Nicotinic acid supplementation (NA) causes a greater increase in glucose and HbA1c levels than nicotinamide (NE) supplementation (Supplementary Table).

##### The effect of NAD + precursor supplementation on liver enzymes and CRP

**Fig. 3:**
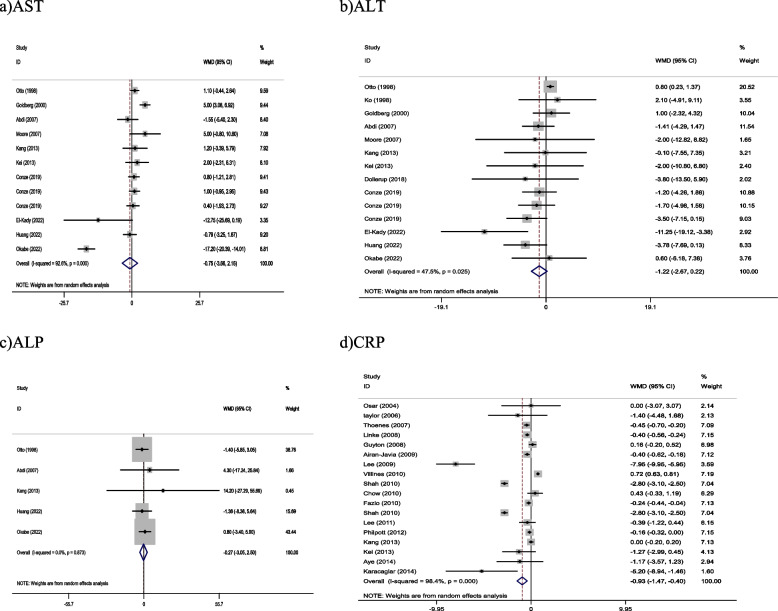
Forest plot of randomized controlled trials investigating the effects of NAD + precursor supplementation on (**a**) AST, (**b**) ALT, (**c**) ALP, (**d**)CRP (mg/l)

Pooled findings from the random-effects model indicated that ALT (WMD: -1.22 U/L, 95% CI: -2.67 to 0.22, *P* = 0.098), AST (WMD: -0.75 U/L, 95% CI: -3.66 to 0.16, *P* = 0.614), and ALP (WMD: -0.27 U/L, 95% CI: -3.05 to 2.50, *P* = 0.846) were not significantly changed after NAD + precursor supplementation compared to control group. Howeve, NAD + precursor supplementation significantly reduced CRP (WMD: -0.93 mg/l, 95% CI -1.47 to -0.40, *P* < 0.001) levels compared to the control group. Furthermore, a significant heterogeneity was found among the studies for CRP (Cochran *Q* test, *P* = 0.002, I^2^ = 96.5–99.2%), ALT (Cochran *Q* test, *P* = 0.025, I^2^ = 44.2–49.9%) and AST (Cochran *Q* test, *P* < 0.001, I^2^ = 92.6%), but a low heterogeneity was reported for ALP (Cochran *Q* test, *P* = 0.873, I^2^ = 0.5–0.9%; Fig. 3).

#### Subgroups analysis

The findings of the subgroup also show the greater effect of NAD + precursor supplementation on ALT increase in a duration of intervention ≤ 12 weeks. In addition, subgroup analysis showed that the increase in ALT was greater when receiving supplementation NE than NA supplementation (Supplementary Table). The reducing effect on CRP concentration was also greater in the dose of ≥ 2 g, the duration of the intervention was ≤ 12 weeks, and with the supplement of NA (Supplementary Table).

#### *Meta*-regression

Meta-regression between NAD + precursor and absolute mean differences in CRP, ALT, AST, glucose, insulin, HOMA-IR, and HbA1c based on dosage and duration of intervention was performed. Only, there was a significant relationship between duration of intervention with changes in ALT (coefficient (Coef) = 0.1589788, *P* = 0.004). However, meta-regression analysis not showed a significant linear relationship between dose and duration of intervention with changes in other variables (Supplementary Figs. 1–7).

#### Sensitivity analysis

We gradually removed each trial from the analysis in order to determine the impact of each article on the aggregated effect size for the levels of glucose, insulin, HOMA-IR, HbA1c, CRP, AST, ALT, and ALP. The robustness of the findings was demonstrated by the leave-one-out sensitivity analysis (Supplementary Figs. 8–9).

#### Publication *bias*

Based on the Egger's tests, no indication of publication bias was found for the following variables: glucose (*P* = 0.762), insulin (*P* = 0.788), HOMA-IR (*P* = 531), HbA1c (*P* = 436), CRP (*P* = 0.970), AST (*P* = 0.131), ALT (*P* = 0.622), and ALP (*P* = 0.327) (Supplementary Figs. 10–11).

## Discussion

Our comprehensive review and meta-analysis revealed that supplementing with NAD + precursors increased blood glucose and HbA1c in humans much more than placebo or no therapy, but that it had no statistically significant effect on insulin or HOMA-IR. To the best of our knowledge, no meta-analysis has been done on the impact of NAD + precursors on healthy and other papulation people' glucose metabolism. The effects of nicotinamide adenine dinucleotide (NAD +) precursor supplementation on glucose and lipid metabolism in humans were examined in the meta-analysis carried out by Zhong et al. Only studies that allowed diabetes and works of English literature, however, were included [21]. Earlier meta-analysis investigated and described the effect of NAD + precursor supplementation on improving TG, TC, LDL, and HDL levels in humans, but resulted in hyperglycemia, compared with placebo or no treatment [21, 79]. Animal studies evaluating obese mice have shown an association between NAD + supplementation and improved indices of obesity as well as molecular regulation of adipocytes [80, 81].

To evaluate these results, it is worth noting that NAD + is an important molecule in energy and signal transduction, in addition to acting as a substrate for enzymes such as sirtuins, poly-ADP ribose polymerases (PARPs) and cyclic ADP ribose synthetases that regulate cellular processes key to energy metabolism, DNA damage repair, and calcium signaling [82]. And the relationship between NAD + precursor supplementation and increased blood glucose can be explained by the function of NAD + in pancreatic beta cells, responsible for insulin production [83]. Insulin is a hormone that regulates blood glucose levels, allowing cells to absorb glucose from the blood and use it as energy, a function that is increased by the use of intracellular NAD supplementation, as mentioned by Reimers et al. [84]. According to Yoshino et al. [85] NAD + supplementation can lead to increased insulin production by pancreatic beta cells resulting in increased glucose absorption by body cells.

Still, other important functions that could explain the significant increase in glucose and HbA1c in humans compared to placebo or no treatment, is that nicotinamide has the ability to scavenge free radicals, as well as provide protection against toxic stimuli and against depletion of intracellular NAD. However, when their levels are still high, as in the case of supplementation or even by endogenous pathways, they are able to inhibit NAD-dependent functions, causing an increase in glucose metabolism and preventing the process of aerobic glycolysis, consequently generating an increase in glucose [86, 87].

Thus, it can be evaluated that the insulin response and HOMA-IR tend not to present significant results, as occurred in this study, since this information regarding supplementation can generate conflicts, depending on the amount in the body of each individual, being a limiting factor of the response to supplementation.

NAD + supplementation may also affect glucose production in the liver. NAD + is required for the proper function of several hepatic enzymes involved in glucose metabolism, including gluconeogenesis, the process by which the liver produces glucose from non-glidic precursors. NAD + supplementation may increase the activity of these hepatic enzymes, resulting in an increase in glucose production by the liver [25]. However, we found no systemic changes in ALT, AST, or ALP levels after NAD + precursor supplementation when compared to the control group.

The biochemical regulation of nicotinamide in the blood takes place through hepatic regulation, involving its conversion to stored NAD through hydrolysis or the reverse cycle. This reaction helps maintain NAD levels within normal limits [88]. Therefore, in order to observe changes in liver enzymes, very high doses must be administered and controlled to avoid generating hepatotoxicity. However, the results of NAD + supplementation on liver enzymes did not change due to such factors. Additionally, the study duration was not sufficient to produce evident effects.

In this meta-analysis, we look at the relation between supplementation and HbA1c, or glycosylated hemoglobin, which is a test used to measure the average blood sugar level over the past 2–3 months [89]. It is formed when hemoglobin, a protein in red blood cells, binds to glucose in the bloodstream. The amount of HbA1c in the blood can be used as an indicator of how well a person's blood sugar has been controlled over time, which is important for managing diabetes. HbA1c and NAD + can be important for maintaining overall health and wellness. Proper blood sugar control is crucial for managing diabetes and reducing the risk of complications, while NAD + plays a vital role in cellular energy production and DNA repair. Given the evidence of supplementation and increased glucose and HbA1c, it is important to emphasize that NAD + supplementation should not be seen carried out in pathological settings because an increase in blood glucose is undesirable in some conditions, such as diabetes, and, as a result, NAD + supplementation should be carried out under appropriate supervision [90].

With respect to the significant reduction in CRP concentration in treated individuals, our findings showed a potential anti-inflammatory effect with NAD + precursors supplementation. Although the exact mechanism to explain this relationship has not yet been established, it is suggested that the reduction in CRP concentration in individuals supplemented with NAD + precursors, such as NA, may also be related to its effects as lipid lowering agents [91].

Our results showed that the magnitude of the reduction in CRP concentration was also greater with a dosage equal to or greater than 2 g of NAD + precursors. However, contrary to what was observed for BP, the effect was more expressive with a treatment time equal to or less than three months. Such findings suggest that for acute biochemical parameters of cardiovascular importance, such as CRP, a treatment with high doses but with a shorter duration is more effective.

Our study had some limitations that jeopardized the extraction of robust conclusions. Clinically and statistically significant heterogeneities was found for adiponectin. These may be explained by the differences in the intervention-specific factors (e.g., type, dose, administration route, and duration of drugs) and blood pressure/inflammation-specific factors (e.g., age, sex, physiology, genetics, familial history, race/ethnicity, physical activity, socioeconomic status, dietary intakes, and drug, tobacco, or alcohol consumption) [92]. Nonetheless, we attempted to identify some possible sources of heterogeneity in data by performing a subgroup analysis. As a limitation of this systematic review we only include studies with an intervention duration of more than 4 weeks. We have included this limitation to ensure the validity of the results as well as the quality design of the studies in this meta-analysis study. In addition, lack of registration of the current study in PROSPERO due to time limit was another limitation of this study. Despite its limitations, the current study has several positive features: a rigorous methodology was used based on the PRISMA guidelines [93]; A thorough literature search using multiple independent databases; two researchers independently and in duplicate searched, selected, and extracted data from the selected studies; To resolve disputes, a third party was consulted [94, 95]. Furthermore, the present study likely included the largest effect size for each outcome assessed at glucose metabolism and liver enzymes.

Overall, these findings suggest that NAD + precursor supplementation might have a significant effect on glucose metabolism and CRP but does not appear to have a significant effect on liver enzymes. The results highlight the importance of considering the duration and the type of NAD + precursor supplementation when evaluating its effects on glucose metabolism. Further interventional studies with a major period (> 4 weeks) are needed to clarify the mechanisms of action and potential long-term effects of NAD + precursor supplementation on glucose metabolism and liver enzymes.

### Supplementary Information

Supplementary Material 1.

Supplementary Material 2.

Supplementary Material 3.

Supplementary Material 4.

## Data Availability

Data will not be made available in a public repository as we have not obtained ethical clearance to share data publicly. However, on request from corresponding author data could be provided while maintaining anonymity.
