# Supplementary material for: Changes in glucose metabolism, C-reactive protein, and liver enzymes following intake of NAD + precursor supplementation: a systematic review and meta‐regression analysis
Source: Nutr Metab (Lond). 2024 Jun 24;21:35. doi: 10.1186/s12986-024-00812-0 (PMC11195006; doi:10.1186/s12986-024-00812-0)
Supplement: Supplementary file 1 — Supplementary Material 1. [file 12986_2024_812_MOESM1_ESM.docx]

**Supplemental Table 1-** Meta-analyses showing the effect of NAD+ precursor supplementation on several subgroups (all analyses were conducted using random effects model).

|  | | | | | | | | | | | **Heterogeneity** | |  |  |
| --- | --- | --- | --- | --- | --- | --- | --- | --- | --- | --- | --- | --- | --- | --- |
|  | | | | | | | | | **No. of**  **Treatment arms** | **WMD^1^ (95%CI)** | | ***I^2^* (%)** | | ***P* within group** |
| **Fasting glucose (mg/dl)** | | | | | | | | |  |  | |  | |  |
| ***Duration*** | | | *≤ 12 weeks* | | | | | | 16 | **2.07 -0.22 4.37** | | ***64.7*** | | ***<0.001*** |
|  |  |  | *> 12 weeks* | | | | | | 14 | **2.48 -0.03 4.99** | | ***64.6*** | | ***<0.001*** |
| ***Dosage*** | | | *≥ 2 g* | | | | | | 14 | **2.18 0.09 4.27** | | ***48.5*** | | ***0.022*** |
|  |  |  | *< 2 g* | | | | | | 16 | **2.50 -0.25 5.25** | | ***72.7*** | | ***<0.001*** |
| **Type of intervention** | | | *Nicotinic acid (NA)* | | | | | | 25 | **3.39 1.31 5.47** | | ***64.1*** | | ***<0.001*** |
|  |  |  | *Nicotinamide (NE)* | | | | | | 5 | **-0.01 -0.28 0.26** | | ***0.0*** | | ***0.670*** |
| **Insulin (μU/mL)** | | | | | | | | |  |  | |  | |  |
| ***Duration*** | | | | *≤ 12 weeks* | | | | | 6 | **0.47 -1.68 2.62** | | ***63.8*** | | ***0.017*** |
|  |  |  |  | *> 12 weeks* | | | | | 4 | **2.28 -2.92 7.48** | | ***80.7*** | | ***0.001*** |
| ***Dosage*** | | | | *≥ 2 g* | | | | | 3 | **3.94 -2.37 10.24** | | ***78.0*** | | ***0.011*** |
|  |  |  |  | *< 2 g* | | | | | 7 | **0.02 -2.19 2.23** | | ***71.1*** | | ***0.002*** |
| **Type of intervention** | | | | *Nicotinic acid (NA)* | | | | | 8 | **1.26 -1.00 3.53** | | ***73.3*** | | ***0.001*** |
|  |  |  |  | *Nicotinamide (NE)* | | | | | 2 | **-1.43 -4.80 1.93** | | ***15.9*** | | ***0.276*** |
| **HOMA-IR** | | | | | | | | |  |  | |  | |  |
| ***Duration*** | *≤ 12 weeks* | | | | | | | | 7 | **0.05 -0.35 0.46** | | ***70.1*** | | ***0.003*** |
|  | *> 12 weeks* | | | | | | | | 3 | **1.09 -0.81 2.98** | | ***85.6*** | | ***0.001*** |
| ***Dosage*** | *≥ 2 g* | | | | | | | | 1 | **4.30 1.81 6.79** | | ***-*** | | ***-*** |
|  | *< 2 g* | | | | | | | | 9 | **0.03 -0.32 0.38** | | ***64.8*** | | ***0.004*** |
| **Type of intervention** | | *Nicotinic acid (NA)* | | | | | | | 7 | **0.44 -0.26 1.14** | | ***79.4*** | | ***<0.001*** |
|  |  | *Nicotinamide (NE)* | | | | | | | 3 | **-0.16 -0.43 0.11** | | ***0.0*** | | ***0.402*** |
| **HbA1c** | | | | | | | | |  |  | |  | |  |
| ***Duration*** | | | | | *≤ 12 weeks* | | | | 8 | **0.08 0.01 0.14** | | ***0.0*** | | ***0.961*** |
|  |  |  |  |  | *> 12 weeks* | | | | 12 | **0.12 0.04 0.20** | | ***35.8*** | | ***0.104*** |
| ***Dosage*** | | | | | *≥ 2 g* | | | | 10 | **0.11 0.02 0.20** | | ***39.6*** | | ***0.094*** |
|  |  |  |  |  | *< 2 g* | | | | 10 | **0.09 0.03 0.15** | | ***0.0*** | | ***0.824*** |
| **Type of intervention** | | | | | *Nicotinic acid (NA)* | | | | 15 | **0.12 0.06 0.19** | | ***15.2*** | | ***0.284*** |
|  |  |  |  |  | *Nicotinamide (NE)* | | | | 5 | **0.08 0.02 0.15** | | ***0.0*** | | ***0.497*** |
| **CRP (mg/l)** | | | | | | | | |  |  | |  | |  |
| ***Duration*** | | | | | | *≤ 12 weeks* | | | 8 | **-1.47 -2.78 -0.16** | | ***98.2*** | | ***<0.001*** |
|  |  |  |  |  |  | *> 12 weeks* | | | 10 | **-0.43 -0.89 0.03** | | ***97.2*** | | ***<0.001*** |
| ***Dosage*** | | | | | | *≥ 2 g* | | | 8 | **-1.23 -2.19 -0.28** | | ***98.9*** | | ***<0.001*** |
|  |  |  |  |  |  | *< 2 g* | | | 10 | **-0.72 -1.33 -0.11** | | ***96.8*** | | ***<0.001*** |
| **Type of intervention** | | | | | | *Nicotinic acid (NA)* | | | 17 | **-0.95 -1.49 -0.41** | | ***98.5*** | | ***<0.001*** |
|  |  |  |  |  |  | *Nicotinamide (NE)* | | | 1 | **0.0 -3.07 3.07** | | ***-*** | | ***-*** |
| **AST (U/L)** | | | | | | | | |  |  | |  | |  |
| ***Duration*** | | | | | | | *≤ 12 weeks* | | 7 | **0.29 -0.82 1.41** | | ***12.7*** | | ***0.333*** |
|  |  |  |  |  |  |  | *> 12 weeks* | | 5 | **-1.06 -8.25 6.14** | | ***97.2*** | | ***<0.001*** |
| ***Dosage*** | | | | | | | *≥ 2 g* | | 3 | **4.54 2.86 6.23** | | ***0.0*** | | ***0.455*** |
|  |  |  |  |  |  |  | *< 2 g* | | 9 | **-2.32 -5.71 1.08** | | ***93. 2*** | | ***<0.001*** |
| **Type of intervention** | | | | | | | *Nicotinic acid (NA)* | | 7 | **1.66 -0.75 4.06** | | ***69.9*** | | ***0.003*** |
|  |  |  |  |  |  |  | *Nicotinamide (NE)* | | 5 | **-3.05 -8.42 2.32** | | ***96.3*** | | ***<0.001*** |
| **ALT (U/L)** | | | | | | | | |  |  | |  | |  |
| ***Duration*** | | | | | | | | *≤ 12 weeks* | 9 | **-2.28 -3.74 -0.82** | | ***6.2*** | | ***0.384*** |
|  |  |  |  |  |  |  |  | *> 12 weeks* | 5 | **0.79 0.23 1.35** | | ***0.0*** | | ***0.988*** |
| ***Dosage*** | | | | | | | | *≥ 2 g* | 4 | **0.06 -2.79 2.91** | | ***0.0*** | | ***0.737*** |
|  |  |  |  |  |  |  |  | *< 2 g* | 10 | **-1.51 -3.28 0.26** | | ***61.6*** | | ***0.005*** |
| **Type of intervention** | | | | | | | | *Nicotinic acid (NA)* | 7 | **0.71 0.16 1.26** | | ***0.0*** | | ***0.808*** |
|  |  |  |  |  |  |  |  | *Nicotinamide (NE)* | 7 | **-2.69 -4.52 -0.86** | | ***17.0*** | | ***0.300*** |
| **ALP (U/L)** | | | | | | | | |  |  | |  | |  |
| **Type of intervention** | | | | | | | | *Nicotinic acid (NA)* | 3 | **-1.00 -5.33 3.34** | | ***0.0*** | | ***0.677*** |
|  |  |  |  |  |  |  |  | *Nicotinamide (NE)* | 2 | **0.23 -3.38 3.83** | | ***0.0*** | | ***0.604*** |
