# Supplementary material for: Changes in glucose metabolism, C-reactive protein, and liver enzymes following intake of NAD + precursor supplementation: a systematic review and meta‐regression analysis
Source: Nutr Metab (Lond). 2024 Jun 24;21:35. doi: 10.1186/s12986-024-00812-0 (PMC11195006; doi:10.1186/s12986-024-00812-0)
Supplement: Supplementary file 2 — Supplementary Material 2. [file 12986_2024_812_MOESM2_ESM.docx]

|  |  |
| --- | --- |
| **Supplementary Figures 1**. Meta-regression analysis encompassing Glucose changes according to the duration of intervention (weeks) and dose of intervention (g). | |
|  |  |
| **Supplementary Figures 2**. Meta-regression analysis encompassing Insulin changes according to the duration of intervention (weeks) and dose of intervention (g). | |

|  |  |
| --- | --- |
| **Supplementary Figures 3**. Meta-regression analysis encompassing HOMA-IR changes according to the duration of intervention (weeks) and dose of intervention (g). | |
|  |  |
| **Supplementary Figures 4**. Meta-regression analysis encompassing HbA1c changes according to the duration of intervention (weeks) and dose of intervention (g). | |

|  |  |
| --- | --- |
| **Supplementary Figures 5**. Meta-regression analysis encompassing AST changes according to the duration of intervention (weeks) and dose of intervention (g). | |
|  |  |
| **Supplementary Figures 6**. Meta-regression analysis encompassing ALT changes according to the duration of intervention (weeks) and dose of intervention (g). | |

|  | | |  | |
| --- | --- | --- | --- | --- |
| **Supplementary Figures 7**. Meta-regression analysis encompassing C-reactive protein changes according to the duration of intervention (weeks) and dose of intervention (g). | | | | |
| A)   | B)   | |  |  |
| C)   | D)   | |  |  |

**Supplementary Figures 8**. Sensitivity analysis of the weighted mean difference (WMD) for A) Glucose, B) Insulin, C) HOMA-IR, and D) HbA1c.

| A)   | B)   |
| --- | --- |
| C)   | D)   |

**Supplementary Figures 9**. Sensitivity analysis of the weighted mean difference (WMD) for A) AST, B) ALT, C) ALP, D)CRP

| A)   | B)   |
| --- | --- |
| C)   | D)   |

**Supplementary Figures 10**. Funnel plots for evaluation of publication bias of A) Glucose, B) Insulin, C) HOMA-IR, and D) HbA1c.

| A)   | B)   |
| --- | --- |
| C)   | D)   |

**Supplementary Figures 11**. Funnel plots for evaluation of publication bias of A) AST, B) ALT, C) ALP, D) CRP
