# Supplementary material for: Changes in glucose metabolism, C-reactive protein, and liver enzymes following intake of NAD + precursor supplementation: a systematic review and meta‐regression analysis
Source: Nutr Metab (Lond). 2024 Jun 24;21:35. doi: 10.1186/s12986-024-00812-0 (PMC11195006; doi:10.1186/s12986-024-00812-0)
Supplement: Supplementary file 4 — Supplementary Material 4. [file 12986_2024_812_MOESM4_ESM.docx]

***Supplementary Appendix1.***

***S1. Search strategy***

***PubMed/Medline***

(“NAD"[MeSH Terms] OR "NAD precursor"[Title/Abstract] OR "Nicotinic Acids"[MeSH Terms] OR "Nicotinic Acid"[Title/Abstract] OR "NA"[Title/Abstract] OR "Niacin"[MeSH Terms] OR "Niacin"[Title/Abstract] OR "Niacinamide"[MeSH Terms] OR "Niacinamide"[Title/Abstract] OR "Nicotinamide"[Title/Abstract] OR "NAM"[Title/Abstract] OR "Nicotinamide Riboside"[Title/Abstract] OR "NR"[Title/Abstract] OR "Nicotinamide Mononucleotide"[MeSH Terms] OR "Nicotinamide Mononucleotide"[Title/Abstract] OR "NMN"[Title/Abstract] OR Niaspan[Title/Abstract] OR acipomax [Title/Abstract] OR Niagen[Title/Abstract]) AND (“Glycated Hemoglobin A"[Mesh] OR “Glycated Hemoglobin A”[ All Fields]OR HbA1c[All Fields] OR “Insulin Resistance”[ All Fields] OR “Insulin Resistance”[MeSH] OR Insulin[All Fields] OR Insulin[Mesh] OR Glucose[All Fields] OR “Glucose Intolerance”[ All Fields] OR Glucose[Mesh] OR “Glucose Intolerance”[Mesh] OR AST[All Fields] OR ALT[All Fields] OR ALP[All Fields] OR SGOT [All Fields] OR SGPT [All Fields] OR “Aspartate Transaminase” [Mesh] OR “Alanine Transaminase” [Mesh] OR “Alkaline Phosphatase” [Mesh] OR CRP OR C-reactive protein [Mesh] OR inflammation) AND ("Clinical Trials as Topic"[Mesh] OR "Cross-Over Studies"[Mesh] OR "Double-Blind Method"[Mesh] OR "Single-Blind Method"[Mesh] OR "Random Allocation"[Mesh] OR RCT[All Fields] OR "Clinical Trial" [Publication Type] OR "Controlled Clinical Trials as Topic"[Mesh] OR "Intervention Studies"[ All Fields] OR intervent*[All Fields] OR Trial[All Fields] OR "controlled trial"[ All Fields] OR randomize*[All Fields] OR randomise*[All Fields] OR random*[All Fields] OR placebo[All Fields] OR assignment[All Fields])

***Supplementary Appendix 2:***

***R codes used for analysis:*** ***U and W are Excel columns and we considered R as 0.5 based on studies.***

***SQRT(((U:U)^2+(W:W)^2)-(2*0/5*U:U*W:W))***
